# Modelization of Nutrient Removal Processes at a Large WWTP Using a Modified ASM2d Model

**DOI:** 10.3390/ijerph15122817

**Published:** 2018-12-11

**Authors:** Jakub Drewnowski, Jacek Makinia, Lukasz Kopec, Francisco-Jesus Fernandez-Morales

**Affiliations:** 1 Faculty of Civil and Environmental Engineering, Gdansk University of Technology, 80-233 Gdansk, Poland; jdrewnow@pg.edu.pl (J.D.); jmakinia@pg.edu.pl (J.M.); 2 Ekofinn-Pol Ltd, 80-297 Banino, Poland; lk@ekofinn.pl; 3 ITQUIMA, Chemical Engineering Department, University Castilla-La Mancha, 13071 Ciudad Real, Spain

**Keywords:** ASM2d, batch tests, denitrification, hydrolysis, internal C source, OUR

## Abstract

The biodegradation of particulate substrates starts by a hydrolytic stage. Hydrolysis is a slow reaction and usually becomes the rate limiting step of the organic substrates biodegradation. The objective of this work was to evaluate a novel hydrolysis concept based on a modification of the activated sludge model (ASM2d) and to compare it with the original ASM2d model. The hydrolysis concept was developed in order to accurately predict the use of internal carbon sources in enhanced biological nutrient removal (BNR) processes at a full scale facility located in northern Poland. Both hydrolysis concepts were compared based on the accuracy of their predictions for the main processes taking place at a full-scale facility. From the comparison, it was observed that the modified ASM2d model presented similar predictions to those of the original ASM2d model on the behavior of chemical oxygen demand (COD), NH_4_-N, NO_3_-N, and PO_4_-P. However, the modified model proposed in this work yield better predictions of the oxygen uptake rate (OUR) (up to 5.6 and 5.7%) as well as in the phosphate release and uptake rates.

## 1. Introduction

In the available literature, the potential use of external substrates for the enhancement of the main Biological Nutrient Removal (BNR) processes has been described, paying special attention to the application of soluble and readily biodegradable substrates [1,2,3,4]. Nowadays, the major strategic priorities of European Union (EU) policy strongly support research activities related to the development of innovative environmental technologies for wastewater treatment plants (WWTP). One of the proposed activities is the use of slowly biodegradable internal carbon (C) sources, such as the particulate substrate (X_S_), to enhance the nutrient removal from the wastewaters. The final aim of this activity is to fulfil the regulations imposed to the full-scale WWTPs by the EU Directive [5]. In this work, with the aim to study the effect of these internal C sources over the BNR processes taking place in the WWTP, the process kinetics of the biological phosphorous and nitrogen removal were studied in both: the full-scale WWTP as well as in batch tests carried out with actual wastewaters from the full-scale WWTP. The information obtained from these tests could be used in two ways: to optimize the WWTP, and to provide guidelines for retrofitting the activated sludge reactors currently operated in these plants.

The modeling of the effects of X_S_ at a WWTP implementing BNR processes is complicated due to the fact that municipal wastewater is composed by a complex mixture of particles, colloids, and soluble pollutants with variable compositions and concentrations. Because of that, different chemical oxygen demand (COD) fractions have to be quantified in order to adequately characterize the wastewater for its subsequent use as input data for modeling purposes. Ekama and Marais [6] divided the biodegradable COD fractions of the wastewater into two distinct fractions. The readily biodegradable fraction (S_S_) mainly consists of soluble organic compounds and the slowly biodegradable particulate fraction (X_S_) which consists of particles, colloids, and large molecules. The impact of the S_S_ in BNR processes has been extensively investigated [7], but there is still not enough study of X_S_ as a major part of internal C source in the wastewater on BNR processes. The biodegradation of X_S_ starts by a hydrolytic process usually described in the activated sludge models [3]. This concept of hydrolysis is in use for over 20 years in activated sludge models (ASM) developed by international water association (IWA) Task Scientific Group, but still requires attention because of its relevance in advanced computer simulations platforms. 

According to the literature, in a conventional activated sludge process, the five-day biochemical oxygen demand to nitrogen and phosphorous ratio (BOD_5_:N:P) in order to avoid nutrient deficiency is 100:5:1 [8]. However, depending on the characteristics of the C source consumed, its value could change [9,10]. A widely used experimental approach to study the hydrolysis is to evaluate this process by using different types of batch tests fed with wastewater as a source of internal C source containing different substrates, mainly biodegradable S_S_ and X_S_, and seed the reactor with heterotrophic biomass. Experimental results obtained in these tests can be then evaluated by mathematical modeling and advanced computer simulations to describe the hydrolysis process. Finally, the results obtained should be verified in full scale experiments. Because of that, this work was divided into two stages: a first stage consisting of experimental research, and a second stage consisting on mathematical modelling using advanced computer simulation and actual data from the full-scale WWTP. 

In the first stage of the study, an innovative procedure for the evaluation of the X_S_ fraction over the BNR processes was implemented [11]. The results of laboratory tests were further used in the second stage to evaluate the hydrolysis process using the activated sludge model “ASM2d” [3]. This model takes into account the biological carbon removal (biological oxidation to CO_2_) as well as nutrient removal processes (biological phosphorus and nitrogen removal by means of biomass storage and nitrification-denitrification respectively) including the denitrifying activity of the phosphorus accumulating organisms. Additionally, based on the dual hydrolysis model concept presented in the literature [12], a modified version of ASM2d model was used and evaluated in this work with the aim to study the effective use of internal C source to enhance BNR processes at second largest WWTP in northern Poland, the Debogorze WWTP.

## 2. Materials and Methods 

### 2.1. Full-Scale WWTP

The Debogorze WWTP (54° 34’ 38.3046" N, 18° 25’ 52.8924" E) was expanded and modernized on June 2009 before the studied periods. This facility has four activated sludge reactors, each with a volume of 12,000 m^3^, implementing the Johannesburg BNR process configuration and six secondary clarifiers. The Johannesburg BNR configuration allows the activated sludge to remove nitrogen, by the nitrification–denitrification process, and to the removal of phosphorus, by means of the accumulation inside the biomass as poly-phosphate. The Johannesburg process configuration has a separate anoxic zone for the denitrification of the return activated sludge before it is introduced into the anaerobic compartment. Operating in this way, it is reduced the nitrate load entering the anaerobic compartment which reduced the competence for the organic substrate between the nitrogen and phosphorus removal processes. This operational configuration leads to an enhancement of the biological phosphorous removal yield.

During the fall (September–November) and spring (May) study periods, the Debogorze WWTP operated at temperatures ranging from 15.4–17.8 °C. The mixed liquor suspended solids (MLSS) concentrations was maintained at 4750 g/m^3^ and the sludge age was 29 d. The monthly average concentrations of the main pollutants in the wastewater are presented in Table 1. More details concerning wastewater characteristics and operating parameters from the study plant can be found elsewhere [11,13].

### 2.2. Batch Laboratory Study.

**Figure 1:**
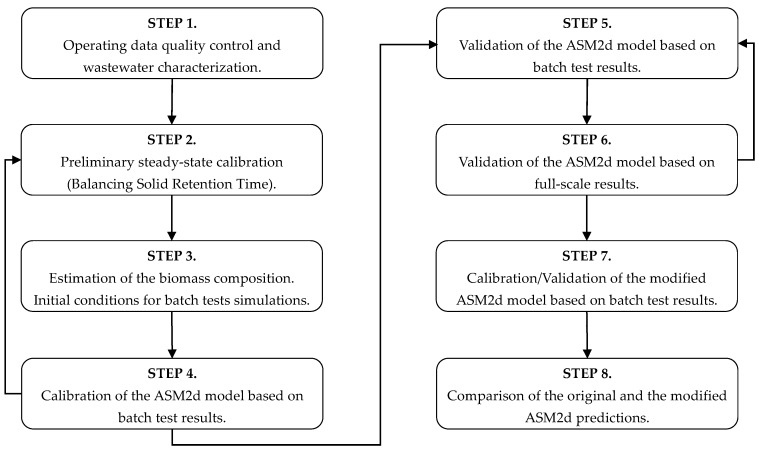
Scheme of the procedure followed in the modelization.

Laboratory tests were carried out by using activated sludge from the bioreactor of the Debogorze WWTP, and actual settled wastewater (SWW) from the average daily time-proportional sampler. According to the procedure described in the literature [15], two kinds of wastewaters were used: SWW without any pretreatment and pretreated with coagulation–flocculation (C–F) method. The wastewater pretreated with the C–F was prepared following the procedure described in the literature [16]. This wastewater only contained the soluble organic fraction. Both wastewaters were used to carry out different types of laboratory batch tests in order to determine the oxygen uptake rate (OUR), ammonia uptake rate (AUR), nitrate uptake rate (NUR), phosphorous uptake rate (PUR), and phosphorous release rate (PRR). The original and the modified ASM2d models were calibrated using results obtained from the batch tests carried out with the SWW [17,18]. The same set of model parameters were further evaluated using steady state data from Debogorze WWTP to compare predictions of the original and the modified ASM2d models at Debogorze WWTP. Organization of the modeling is presented in Figure 1.

**Figure 2:**
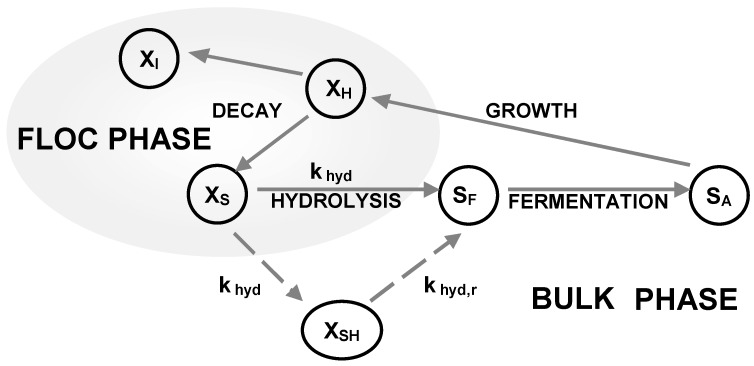
Scheme of the ASM2d, in continuous lines, and the modification proposed in the hydrolysis process, in dashed lines. X_I_: Inert particulate substrate; X_H_: Heterotrophic organisms; X_S_: Particulate substrate; X_SH_: Rapidly hydrolysable substrate; S_F_: Fermentable substrate; S_A_: Fermentation product; K _hyd_: First Hydrolysis stage; K _hyd,r_: Second Hydrolysis stage.

The modified ASM2d used in this work considered a two-step hydrolysis process with two rates (k_hyd_, k_hyd,r_), includes a new component (X_SH_), defined as the substrate readily hydrolysable and three new hydrolysis processes of X_SH_ carried out under aerobic, anoxic and anaerobic conditions. A scheme of the hydrolysis concept is presented in Figure 2.

### 2.3. Analytical Methods

The wastewater characterization was carried out according to the standard methods [19]. Most of the total and soluble fractions of the COD, nitrate and phosphate were characterized by using a Xion 500 spectrophotometer (Hach Lange GmbH, Düsseldorf, Germany). Only the total nitrogen concentration was determined by using a TOC/TN analyzer (SHIMADZU Corporation, Tokyo, Japan). The gravimetric analyses also were performed in accordance with the Standard Methods procedure [19].

## 3. Results and Discussion

**Figure 3:**
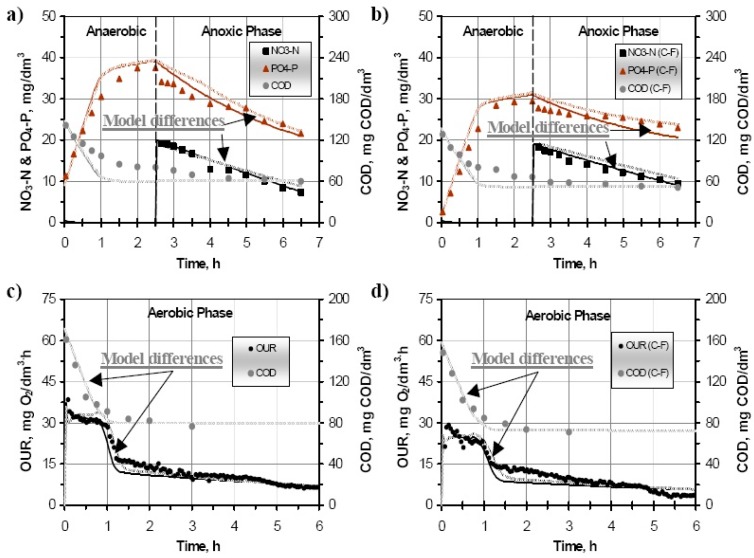
Experimental data and model predictions in the batch test treating SWW without pretreatment and after C–F. Solid lines corresponds to conventional ASM2d predictions and dashed ones to the modified ASM2d predictions. (**a**) PRR/anoxic PUR tests without pretreatment; (**b**) C–F treatment for PRR/anoxic PUR tests; (**c)** OUR tests without pretreatment; (**d**) C–F treatment for OUR tests.

The application of the C–F method removed the colloidal and particulate COD fractions from the SWW. This removal resulted in processes rates reduction ranging in most of the cases from 10–60%. Similar results were previous obtained and reported in the literature [11]. The comparison of both examined models’ predictions vs. sample results of principal processes are presented in Figure 3. The ASM2d predictions were calibrated to the experimental NURs tests by fitting two parameters: the maximum growth rate of heterotrophs (μ_H_) and hydrolysis rate constant (k_h_). No further fitting were required to calibrate the NUR in the anoxic stage of the PRR/anoxic PUR test (Figure 3a,b). Six parameters were fitted to calibrate the PRR and PUR tests. These parameters were the rate constant for storage of PHA (q_PHA_), half saturation coefficient of S_A_ for PAOs (K_SA, PAO_), polyphosphate saturation coefficient for PAOs (K_PP_), the reduction factor of the anaerobic hydrolysis (η_fe_), and particulate COD saturation coefficient (K_X_). The nitrogen removal process, based on the PRR and the PUR batch tests, was calibrated fitting the maximum growth rate of autotrophs (μ_A_) and the NH_4_-N saturation coefficient (K_NH4,A_). The critical step of OUR batch test fitting was to adjust average values of both stoichiometric and kinetic parameters in the modified ASM2d. To do this fitting, 10 different scenarios were evaluated. The initial ARD were determined, being the ARD of the COD profile lower than 15% in all the cases, whereas higher ARD were obtained when predicting the OUR values which presented values up to 45%. The very high ARD in the OUR parameter could be explained because of the low consumptions rates, about 0.02 g g^−1^ VSS h^−1^, which lead to high ARD even when the absolute variations were small [20]. In order to reduce these errors, the OUR predictions were optimized by using the Nelder–Mead method [21]. Finally, using equal sets of model parameters from previous batch tests, the modified ASM2d were further evaluated by steady state and dynamic simulations of OUR batch test (Figure 3c,d).

From these tests, the values of the hydrolysis rate constants (k_hyd_ and k_hyd,r_) of the two step-hydrolysis model proposed in the modified ASM2d model were mathematically determined, being their values 2.0 and 10 d^−1^, respectively. In the case of the conventional ASM2d model the single hydrolysis rate constant was 2.5 d^−1^. The very different hydrolysis rate of the particulate slowly biodegradable substrates, k_hyd,r_ 10 d^−1^, indicates that two different fractions can be identified as products of the hydrolysis stage. Because of that, the mechanism of the hydrolysis stage is better described by taken into account two separately rates. When identifying these two stages of the hydrolysis, the model predictions of the COD and nutrient profiles were more accurate. This higher accuracy in the nutrient profiles can be explained because the nutrient removal rates and yields clearly depends on the nature of the substrate used [22,23]. In this way, the use of the modified ASM2d model proposed in this work is an interesting option to obtain more accurate predictions at WWTP. These better predictions were expected in all the biological processes taking place in the WWTP, but a more significant effect on the nutrient removal processes was observed, mainly in the phosphate removal, because the organic substrate and the subsequent poly-phosphate accumulation inside the biomass is very sensitive to the characteristics of the organic C source used [24]. 

Concerning the stoichiometric parameters, they were very similar in all the cases. In the case of the heterotrophic biomass yield (Y_H_) obtained in the modified ASM2d model was 0.68, being the corresponding value in the original ASM2d model slightly higher, 0.625. The similar results obtained when using both models, indicates that the model modifications, carried out in the modified ASM2d model proposed in this work, did not significantly influence the stoichiometric of the processes.

The predictive capabilities of the original and modified ASM2d have been confirmed by ARD, which were much lower for the simulation with the modified ASM2d. For comparison, the ARD for the original vs. modified ASM2d simulation of the OUR tests accounted for 11.3–29.5% and 18.9–45.8% vs. 9.7–15.8% and 11.8–30.3% for the settled wastewater without pretreatment and after coagulation–flocculation, respectively. As a summary, the most relevant stoichiometric and kinetic coefficients values in the original and the modified ASM2d models are presented in Table 2.

After the calibration step, the quality of the predictions of the original and modified ASM2d were determined by evaluating the average relative deviation (ARD). Table 3 contains the ARD obtained with the original and the modified ASM2d models in the main processes evaluated with the SWW with and without the C–F pretreatment at Debogorze WWTP.

As can be seen in Table 3, the very similar results were obtained in both models when evaluating the COD consumption, phosphate release, and ammonia utilization whereas the largest one were found for nitrate utilization, up to 9.6%, phosphate uptake, up to 11.3%, and oxygen uptake, up to 5.7%. These results indicate that the modified model yields more accurate predictions.

## 4. Conclusions

In the present work, a modified ASM2d model, based on a novel two stage hydrolysis concept, has been evaluated. From the obtained results, the following conclusions can be extracted:

- The modified ASM2d model presented in this work allows reaching more accurate predictions of the behavior of the activated sludge systems taking place in a full scale WWTP than the original ASM2d model. Additionally, more accurate assessment of wastewater biodegradability in terms of the COD fractions was obtained which is crucial for the BNR processes modelization.
- When comparing the original and the modified ASM2d models it was observed that the largest differences in the ARD values were obtained in the predictions of nitrate utilization, up to 9.6%, phosphate uptake, up to 11.3%, and oxygen uptake, up to 5.7%.
- When comparing the original and the modified ASM2d only minor effect were observed on the behavior of COD consumption, phosphate release, and ammonia utilization.
- The effective use of internal C sources, such as slowly biodegradable substrate (X_S_) for denitrification and biological phosphorous removal may help to reach the quality standards stablished in the EU regulations for large WWTPs.
- From the modelling results, it was observed that the colloidal and particulate organic fractions play a crucial role the enhancement of the denitrification and EBPR at the Debogorze WWTP.

## Figures and Tables

**Table 1:** Characteristics of the influent wastewater during studied periods at Debogorze WWTP.

Definition	Symbol	Unit	Monthly Average Value	Source of Data
Influent COD	COD_in_	g COD/m^3^	856	Laboratory analyses
Influent COD in filtered sample	COD_f,in_	g COD/m^3^	211	Laboratory analyses
Volatile Fatty Acids	VFA	g/m^3^	167	Laboratory analyses
Influent BOD_5_	BOD_5,in_	g BOD_5_/m^3^	319	Laboratory analyses
Influent Biodegradable COD	BCOD_in_	g COD/m^3^	545	Calculation [14]
Effluent COD	COD_out_	g COD/m^3^	25.4	Laboratory analyses
Effluent COD in filtered sample	COD_f,out_	g COD/m^3^	20.5	Laboratory analyses
BOD_5_/BOD_U_ ratio	fBOD	–	0.67	Laboratory analyses

WWTP: Wastewater treatment plant; COD: Chemical oxygen demand; BOD_5_: Five days biochemical oxygen demand; BOD_u_: Ultimate biochemical oxygen demand.

**Table 2:** Comparison of the calibration values at the original and the modified ASM2d.

Symbol	Unit	Original ASM2d	Modified ASM2d
***Stoichiometric coefficients***			
Y_H_	gCOD/gCOD	0.625	0.68
***Kinetic coefficients***			
k_hyd_	1/d	2.5	2
k_hyd,r_	1/d	-	10
η_fe_	-	0.1	0.1
η_fer_	-	-	0.4
k_x_	1/d	0.2	0.1
k_xr_	1/d	-	0.03
η_NO3, Hyd_	-	0.6	0.6
η_NO3, Hydr_	-	-	0.4
K_O2_	g O_2_ /m^3^	0.2	0.2
K_NO3_	g N/m^3^	0.5	0.5

**Table 3:** Differences in the ARD obtained with the original and modified ASM2d.

Test	Process Rate	ARD Differences between the Original and Modified ASM2d [%]			
		Settled Wastewater		Coagulation-Flocculation	
		Fall	Spring	Fall	Spring
**NUR**	Nitrate utilization	1.6	17.0	1.6	5.0
	Soluble COD utilization	0.3	0.4	0.2	0.0
**PRR/anoxic PUR**	Phosphate release	0.0	0.5	0.3	0.6
	Phosphate uptake	2.2	0.6	3.3	1.1
	Nitrate utilization	9.6	9.0	6.8	1.0
**PRR/aerobic PUR**	Phosphate release	0.5	0.1	0.8	0.1
	Phosphate uptake	5.9	1.9	11.3	4.4
	Ammonia utilization	0.2	0.3	0.4	0.2
	Oxygen uptake	5.4	5.6	1.5	1.2
**OUR**	Oxygen uptake	0.8	0.7	5.7	4.4
	SCOD utilization	0.3	0.4	0.2	1.1

NUR: Nitrate uptake rate; PRR: Phosphorous release rate; PUR: Phosphorous uptake rate; OUR: oxygen uptake rate.
